# Tobacco from an Esthetic and Hygienic Point of View

**Published:** 1896-08

**Authors:** H. M. Ochiltree

**Affiliations:** Haddam, Kas.


THE
AMERICAN JOURNAL
—OF—
DENTAL SCIENCE.

VOL. XXX. BALTIMORE, AUGUST, 1896. NO. 4.

ARTICLE I.

### TOBACCO FROM AN ESTHETIC AND HYGIENIC POINT OF VIEW.

BY H. M. OCHILTREE, M. D., HADDAM, KAS.

Tobacco: one of the curses of the world; as pronounced in its malignant influence as dirt; not so hoary-headed with age as filth, but considered more respectable; only the proverbial pack of dirt, the amount alloted to each individual, but no limit placed on the amount of tobacco used; the average chewer consuming 900 pounds of the weed, causing an expectoration of about 45 barrels of saliva; the average smoker consuming 73,000 cigars, the smoke aacending from the cigars and pipes of the world rivaling the fumes of the bottomless pit.

Be he ever so careful, the tobacco habitue usually presents to the sense of sight or smell the evidence of its use. He deforms his cheek with the cud, blackens his teeth and stains his lips with the juice, and the expectorated filth streaks the corners of his mouth, lodges on his mustache, and stains his clothing; the cigar and pipe disfigures his natural appearance, he smells bad, and, by the authority of the cannibal, he tastes bad, and is a travesty on the civilization of the nineteenth century.

Tobacco was discovered by a civilized race in the fifteenth century. Its use at that time was common among the aboriginal Americans. After a careful study of the anthropological and esthetic nature of the barbarians, our ancestors rejected all manners and customs of the untutored savage but the tobacco habit; this they adopted—a kind of mouth-clout as it were; as a compromise with the breech-clout.

Tobacco produces smoke, odor, filthiness, spit and disease; a quintette of evils arising from no other single cause. Who has not been disgusted by the putrid emanations from the mouth of the chewer, or disseminated into the atmosphere by the smoker? The practice of spitting and smoking in public is a great annoyance, and wherever we go this nuisance is encountered. It is said by one author that a few years of servitude almost annihilates the gentleman; the smoker learns to think of himself alone, and ignores the possibility of offending others by constraining them to inhale the naseous fumes.

The chewer finds it impracticable to be even tolerably clean in its use; the amber must either go down or out, and it generally goes out. A resolute effort is frequently made by a particular chewer to retain the offensive fluid in his mouth when no spittoon, wood-box, fire-place or open window is in sight; but the juice rapidly accumulates; he tips back his head like a chicken when it drinks to keep the amber from overflowing its banks; if in conversation, his words become mere monosyllables, and more guttural; finally utterance fails; spit he must or die; a break is made for the door, and his mouth emptied of one or more ounces of nauseating tobacco-juice.

Some are not so particular, but will stoop to almost any degree of nastiness, and use the floor, hall, mat or pew in a church as a spittoon.

Mackey, the poet, was justly disgusted in his journey through our country by the everlasting spitting going on everywhere. When in Washington, an affable member of Congress was talking with him about our great men. After replenishing his mouth with a big chew of plug tobacco, he asked the poet, "Whom do you consider our greatest general?" The answer was, "General Spit."

There can be no more glaring disregard of decency than that exhibited by expectoration upon the floors in buildings and vehicles used by the public. Measures should be taken to prevent this nuisance—a nuisance not alone because it is disgusting, but because it is unsanitary and in direct opposition to the great law of cleanliness now advocated, if not practiced, by all physicians.

What are the pernicious effects of this ubiquitous article of commerce on the health of its patrons? This is a question of vital importance, and one which physicians, the custodians of health, can not disregard without being guilty of gross neglect in the performance of their sacred duties. Would it not be presumptuous to attempt to inform physicians of the evil effects of tobacco on health? Would any one dare to assert that the learned disciple of Æsculapius who scrutinizes with microscopic eye the Loffler bacillus and other pathogenic germs in his mouth, would with his microscopic eye fail to discover a large quid of unhealthy tobacco in said mouth? Pardon me if I misquote a passage of Scripture: "Thou froward man; first cast the macrococcus (quid) out of thine own mouth, and then thou shalt see more clearly to cast the micrococcus out of thy brother's mouth." Physicians are aware, no doubt, of the evils attending the tobacco habit; its proportions are too Cyclopean to be overlooked. It is the size and strength of the octopus, and the apparent uselessness of making an attack upon the monster, that holds in its slimy tentacles too many members of the medical profession, that prevents a united attack upon this enemy Hygeia.

My hope is to aid, even if it be but little, in the gradual and healthful eradication of this prince of evils, and to render some assistance in opposition to a common enemy. With this object in view it will be opportune to notice briefly some of its undisputed pernicious effects upon the human organism.

The poison in tobacco is an alkaloid called nicotine. Each pound contains about 300 grains, a lethal dose for over 200 men of a poison which resembles hydrocyanic acid in the virulence of its action.

Nicotine is rapidly absorbed by and is a source of irritation to mucous membrane, producing sponginess of the gums, irritation of the buccal cavity, nasal and post-nasal fossa, fauces and larynx, impairing the sense of taste and smell, and producing functional disturbances of the lungs by its poisonous action on the pneumogastric nerve.

All physicians are familiar with the disturbances of digestion caused by its use, due in part to the abundant expectoration of saliva; the waste of saliva is a violation of natural law, but swallowing it more or less saturated with poisonous nicotine is still a great deal worse, and disordered digestion the sequel.

Impairment of digestion results in loss of flesh and strength. J. W. Seaver, of Yale, gives particulars of the comparative condition of a number of students who were moderate users and non-users of tobacco. In weight, height, chest girth, lung capacity and muscular development the percentage of increase was greatly in favor of non-users; thus showing practically the deleterious influence of even the occasional use.

The heart comes in for a large share of mischief. Tobacco acts in some upon the motor and in others upon the sensory nerves of the heart; causing neuralgia, palpitation or tachycardia. Insurance policies contain this question' "To what extent do you use tobacco?" because it is well known that its influence on the heart and other organs is such that it may be the balance of power of evils that cause the final rejection of the applicant.

Cases of epithelioma starting from the mouth in which tobacco is almost continually held is a significant fact. It is well known that locally it will cause irritation of the lips, tongue, gums, mucous membrane of the mouth, cheeks, tonsils, velum and pharynx.

The connection between the use of tobacco and impaired vision is sufficiently obvious. In studying the literature on this subject we find that it is the opinion of distinguished observers that it will cause amblyopia and amaurosis, and that, too, when tobacco is the only narcotic used. Dr. A. R. Parker says: "There is a toxic amblyopia due to excessive use of tobacco different from that caused by the use of stimulants."

The brain and the nerves are already affected by its use, causing impairment of memory, tremor of the hand, cowardice, languor and hypochondriac delusions. Lewin says: "Nicotine psychoses are said to affect smokers and chewers. The prodromal stage shows general uneasiness, restlessness, anxiety, sleeplessness and mental depression; then follows precordial anxiety and finally the psychoses proper."

Bremmer, in a paper on "Tobacco and Insanity," takes the view that it may be a factor in the production of insanity, and in aggravating an existing mental derangement.

Dr. William A. Alcott quotes the German periodicals as stating, "that deaths occurring among men in that country, between eighteen and thirty-five years of age, one-half die from the effects of smoking. They unequivocally assert that tobacco burns out the blood, the teeth, the eyes and the brain."

All are agreed as to its pernicious influence in youth. In schools, non-smokers get on better than smokers; those who smoke showing less intelligience, laziness and a craving for strong drink. One late writer says: "In youth it stunts the growth of the man in all his senses, mentally, morally and physically, acting chiefly by checking oxidation and elimination."

Limitation of time forbids anything but the mere mention of its deleterious effects on the sexual organs, and that it is recognized as a factor in the production of sexual debility, impotence and syphilis. The London *Lancet* says: "No smoker can be a well man."

There is no escape when the laws of Nature are violated by the tobacco habitue; sooner or later, serious injury will follow. Numerous authorities could be quoted to show its baneful effects on all of the senses, on all of the organs of the body, and that no part of the cutaneous, nervous or muscular system escapes this withering curse. Nicotinism, in some of its Proteau forms, will eventually develop in all who persist in the use of tobacco. Let no smoker or chewer flatter himself that he is sound or secure because he feels no harm. All physicians know how frequently men, even in the prime of life are cut down without a moment's warning. Tobacco in many of these cases is no doubt an etiological factor that deserves more than a passing notice.

Kemff says: "A man may lay the foundation for the tobacco habit in his offspring."

Morel, the great investigator of degeneracy, says: "A race which is regularly addicted to tobacco begets degenerate descendants, who, if they remain exposed to the same influence, rapidly descend to the lowest degree of degeneracy."

A pure stream can not spring from an impure fountain; neither can a strong race spring from one whose vital resistance is weakened, The transmitted influence of tobacco on posterity can not be otherwise than harmful.

The contamination of the blood from any source, by any poison, whether by polluted water or nicotine, should receive our earnest consideration. Permit me to ask who is in the most danger, the healthy lad who drinks a typhoid bacillus, or the lad who smokes his first cigar, or taken his first chew of tobacco? Which can claim the most victims, nicotine or the typhoid bacillus?

Well do we know that familiarity with tobacco filth has created a shameful tolerance of it that would not be endured in any other nuisance. It is frequently the case that the first odor that greets the nostrils of the newborn babe is the fumes of tobacco smoke; the infant grows up in an atmosphere charged with tobacco smoke; they see their fathers, brothers, friends and family physician using it, and the natural tendency to imitate and the importunities of unwise friends cause the first step to be taken along a pathway of shame and venomous odors, that leads to the dungeons of galling servitude.

Can anything good be said of tobacco? What use have we for it, if any? It does not occupy a respectable place in therapeutics. In them it is prescribed as a remedy, and a warning given of its evil effects. "It possesses germicidal properties," the devotee exclaims in defense of his favorite narcotic, and for this reason smoking is forbidden in the experimental laboratories. We admit the claim. Fumigate a room with tobacco smoke, and all kinds of life in it would perhaps be destroyed. But the important question is: Is there any practical application of its germicidal properties? Can it be applied in any manner, externally or internally, to cure or prevent cholera, typhoid, diphtheria or any other germ diseases? We have searched in vain for a favorable answer to this injury and although the literature on tobacco is voluminous, not a word do we find in favor of its practical application as a germicide.

A popular idea prevails among the laity that the use of tobacco prevents the entrance of diseased germs into the system. Even if this were true. it would keep one smoking or chewing all the time. One could neither eat nor drink for fear of swallowing a microbe. Malignant diphtheria caused the death of a colleague who smoked almost constantly, smoked from ten to twenty cigars daily. Tubercle bacilli, wrapped in tobacco leaves, retain their virulence up to the tenth day.

Those who use tobacco contract all kinds of diseases, and the theory that it will immunize the habitue is unsupported by any practical demonstration. George M. Powell has shown by the testimony of eminent physicians and navigators that tobacco does not prevent contagious diseases, as many suppose, but that its non-use is actually a condition of safety.

What can we do to prevent the ravages of the tobacco scourge? Permit me to offer for your earnest consideration a few suggestions bearing upon the exigencies of this question. As members of the medical fraternity it is our duty to array ourselves in solid opposition to all enemies of health, practically as well as theoretically.

No one should be granted a certificate to teach in a public school who uses tobacco in any form. The law in this State already prohibits its sale to minors under sixteen years of age, and this law should be supplemented by one forbidding its use by their teachers, who should be required to instruct the pupil of its baneful effects upon the system. The beneficient effects of opposition to its use are already shown by laws passed in several States interdicting its sale to minors under sixteen years of age. These laws should be enforced and strengthened by others—procuring the enactment of laws forbidding its use in public; making it a misdemeanor to smoke in the street or squirt offensive tobacco juice in public places. Medical societies should place the seal of condemnation upon the tobacco habit by countervailing resolutions, and all colleges should require students while under their charge to abstain from its use. Physicians should discourage the habit; one who chews or smokes tobacco can not be consistent. It is certainly a travesty on hygiene to attempt to enforce a law of health that the physician habitually violates; physicians should expurgate themselves of the tobacco habit; the perpetuation of which they are guilty of encouraging by its use; a course that is inconsistent, productive of evil and utterly antagonistic to the principles they teach.

It is said that tobacco is not injurious if used in moderation, and it is used in moderation so long as it is not injurious. A round-about way of saying it is not injurious and a suggestion potent for evil influence, far-reaching in its effects. What is moderation in the use of tobacco ? If a man chews or smokes during a greater part of the day, and eliminates during the night all of the nicotine absorbed during the day, is that moderation ? Is it moderation to take a narcotic posion in any quantity into the system, day after day, and year after year, even though the quantity introduced was eliminated before more of the poison was taken ? "Moderation" in the use of tobacco is a word that has done incalculable harm; nearly all who use the weed excuse themselves by repetition of the term "moderation." This word can not be legitimately applied to the promiscuous use of any poison. Can anything be done by the physician to arrest the progress of the tobacco habit ? Does the tobacco habit come within his scope of practice ? While all must confess to a lamentable degree of impotence to arrest the progress of this pernicious habit, yet it certainly comes within the province of the physician, as we all recognize in the evil effects of tobacco the disease called in medical nomenclature "nicotinism." The plain duty of the physician being to cure and prevent disease, nicotinism must be included in this list, and logically the tobacco habit, its cause. Of the means of relief adopted for the cure of nicotinism, abandonment of the cause will bring about recovery in most cases without any medication. Advice to this effect is often given, but rarely followed, even when supplemented with a warning of the evil effects of tobacco. The diseased nerves, like clamorous demons, call loudly for their accustomed sedative, and the will, enfeebled by long years of servitude, abjectly yields to the exultant enemy. The earnest desire often expressed by many of these unfortunate victims to abandon a habit ignorantly contracted in youth, coupled with an expression of their inability to do so by their own efforts alone, appeals to us to afford, if possible, the desired relief.

Allow me briefly to offer you the results of my own experience with the tobacco habit. I do not offer you a theory, but a practical method that has stood some test. During the last four years I have treated fifty-eight cases of this habit, with 45 per cent. of permanent cures—including none as cured who did not refrain from tobacco in all its forms for at least one year. This percentage, which would be increased as the method of treatment improved, is 15 per cent. greater than that secured by the most approved manner of treating the opium habit. The rationale of my method of treatment is as follows: Tobacco in all its forms must be promptly abandoned. A tonic suitable to each case should be given, and diuretics; laxatives and baths prescribed for the elemination of the poison. My experience teaches me that the habit of holding tobacco in the mouth is difficult to overcome without offering something in the way of an agreeable substitute to replace it. The moral effect of taking something to replace the loss of tobacco is of great value. This substitute should be made in soluble tablet form of from ten to fifteen grains each of some harmless ingredients, and the habitue directed to place a tablet in his mouth whenever the desire for tobacco came on, and directed to continue its use until all desire for tobacco ceased; the tonic to be continued until it met the necessary indications. Some form of substitute must be available at all times during the first year to prevent relapses, which rarely occur after one year's abstinence. No habit is formed by using a carefully selected substitute, which, on the contrary, is voluntarily abandoned during the first year.

The physician will find in the treatment of the tobacco habit, that the gratitude of his patient is as great as when of other serious maladies, and that he is rendering an additional service that will honor him, his profession and posterity.

Permit me in closing to offer an earnest appeal to the medical fraternity. The tobacco habit is hoary-headed with four centuries of years, yet no reasonable defense can be offered for its use. Instead of being the ally, it is the enemy of the physician; yet, to our humiliation be it said, many of us embrace the enemy we should wage an incessant warfare against. Could the goddess Hygeia materialize, and present herself to the smoking, chewing and snuffing physicians of the world, would she not rebuke the hypocritical devotees who worship at her shrine, and yet brush away the glowing tints of health they fain would paint, with another dipped in the poison of the Stygian lake? Would she not say: "Guardians of health, you have sworn allegiance to my cause; but you can not truly serve the goddess of health, while encouraging and assisting in the magic arts of the god Loki. You see young men, old men, sallow and meatless, with an enemy in their mouths stealing away their vitality; you see mere boys dissipating with pipes, cigarettes and stumps of cigars, their faces flushed with tobacco intoxication; you can even foresee the disastrous consequences of the tobacco habit on the physical and mental characteristics of generations yet unborn. Devotees, reverently regard the sacred vows you took when you espoused my holy cause; purify yourselves of the enemies of Hygeia, and then you can go forth as the harbingers of health, arrayed in solid phalanx against all my foes."

Authors consulted: *American Medico-Surgical Bulletin*; Sajob's Annual; Dr. John Lizan, "On the Use and Abuse of Tobacco;" Dr. Henry Gibbons, "Tobacco and Its Effects;" Dr. Wm. A. Alcott, "Tobacco: Its Effects on the Human System."—*Kansas Medical Journal.*

